# Young at Heart: How Subjective Age Influences Perceptions and Expectations of Older Adults

**DOI:** 10.1093/geroni/igaf122.222

**Published:** 2025-12-31

**Authors:** Amy Gourley, Alison L Chasteen

**Affiliations:** University of Toronto, Toronto, Ontario, Canada; University of Toronto, Toronto, Ontario, Canada

## Abstract

A younger subjective age is often associated with positive health outcomes among adults over the age of 65. However, it is also possible that those who attempt to look or act younger than their chronological age may face backlash given this behavior is in violation of prescriptive stereotypes that serve to maintain hierarchical age group boundaries. The degree to which younger and middle-aged perceivers anticipate violations of prescriptive age stereotypes (i.e., that older adults should act their age) may predict negative evaluations of older adult targets who feel ‘younger than their years.’ The present work examined younger and middle-aged adults’ (N = 678; Mage = 35.8, SDage = 9.56) attitudes and evaluations regarding 65-year-old targets who varied by gender (man, woman) and felt age (65, 45, or 25). Participants also reported their expectations regarding older targets’ potential violation of succession-, consumption- and identity- related prescriptive age stereotypes. Expectations of prescriptive stereotype violation mediated the relationship between older targets’ younger felt age and participants’ ratings of targets’ warmth, competence, and interaction intentions. Specifically, targets who felt younger than their chronological age were expected to violate prescriptive stereotypes, which in turn decreased ratings of targets’ warmth and competence and lessened participants’ willingness to interact with targets. Our findings suggest that a younger subjective age may not always benefit older adults. Specifically, consistent with a Social Identity Threat perspective, younger perceivers’ concerns about preserving strict age group boundaries may explain discriminatory attitudes and evaluations of counter-stereotypical older adults.

